# Oral Administration of *Euonymus alatus* Leaf Extract Ameliorates Alzheimer’s Disease Phenotypes in 5xFAD Transgenic Mice

**DOI:** 10.3390/foods13050682

**Published:** 2024-02-23

**Authors:** Yoonsu Kim, Minjung Cho, Chan Ho Jang, Jeong Soon Lee, Jong-Sang Kim, Jisun Oh, Jinkyu Lim

**Affiliations:** 1 Department of Integrative Biology, Kyungpook National University, Daegu 41566, Republic of Korea; yunsu531@gmail.com (Y.K.); cho981023@gmail.com (M.C.); vision@knu.ac.kr (J.-S.K.); 2 Institute of Agricultural Science and Technology, Kyungpook National University, Daegu 41566, Republic of Korea; cksghwkd7@gmail.com; 3 Forest Environment Research Institute of Gyeongsangbuk-do, Gyeongju 38174, Republic of Korea; ljs7942@korea.kr; 4 School of Food Science and Biotechnology, Kyungpook National University, Daegu 41566, Republic of Korea; 5 New Drug Development Center, Daegu-Gyeongbuk Medical Innovation Foundation, Daegu 41061, Republic of Korea

**Keywords:** *Euonymus alatus*, 5xFAD mouse, Alzheimer’s disease, memory improvement, quercetin

## Abstract

Alzheimer’s disease (AD) is the most prevalent neurodegenerative disease and is frequently characterized by progressive and irreversible impairment of cognitive functions. However, its etiology remains poorly understood, limiting therapeutic interventions. Our previous study showed that the ethanol extract of *Euonymus alatus* leaves (EA) positively affected scopolamine-induced hypomnesia in the normal mouse model by promoting nuclear factor E2-related factor 2 (Nrf2) activation. Herein, we examined whether EA administration could ameliorate major AD phenotypes that are manifested in 5xFAD transgenic mice. Two-month-old mice were orally administered with EA at a dose of 50, 100, or 150 mg/kg body weight/day thrice a week for 14 weeks. We observed that EA administration improved behavioral deficits as assessed by the passive avoidance, Morris water maze, and Y-maze tasks; decreased the plasma levels of pro-inflammatory cytokines, including TNFα and IL-1β; decreased the protein expression levels of inflammatory mediators in the hippocampus; and attenuated histological damage and amyloid beta plaques in the hippocampal region of 5xFAD mouse brain. Interestingly, our data demonstrated that the effectiveness was partially attributed to quercetin, which was noted to be a component of EA. Hence, these findings suggest that a long-term administration of EA could alleviate AD symptoms and delay its progression.

## 1. Introduction

For centuries, some Asian countries have used *Euonymus alatus* (EA), an edible plant, for medicinal purposes in the treatment of several conditions, including cancer, hyperglycemia, menstrual discomfort, diabetic complications, and detoxification [1,2]. The Korean Food Code recognizes the young leaves of EA as registered food material. Some components such as rutin, quercetin (QCT), and kaempferol extracted from *Euonymus* spp. have been reported to exhibit antioxidant activity [1,2,3,4]. Moreover, our previous studies demonstrated that the ethanolic extract of EA leaves can improve learning and memory impairment in scopolamine-treated mice and further promote Nrf2-mediated antioxidative activity in the hippocampal region [5].

Maintaining a balance between oxidants and antioxidants is known to be crucial in the prevention of neurodegenerative diseases [6,7,8]. When this balance is disrupted, either through the overproduction of reactive oxygen species or a deficiency in antioxidants, oxidative stress ensues, causing negative impacts on the brain [9]. In neurodegenerative diseases like Alzheimer’s disease (AD), the activity of antioxidant enzymes is altered, thereby accumulating oxidative damage [10,11,12].

AD is characterized by a progressive and irreversible decline in memory and cognitive abilities with three core pathological hallmarks: amyloid plaque, neurofibrillary tangle of hyperphosphorylated tau, and sustained immune response [13]. However, the precise causes of AD remain elusive, demanding further investigation [14,15,16].

Recent studies suggest that the neuroinflammation accompanying the persistent microglial and astrocyte activation in the brain plays a major role in AD pathogenesis and progression [17,18,19,20]. Neuroinflammation, triggered by various damage signals, including oxidative agents, extracellular amyloid beta (Aβ) plaques, and intraneuronal neurofibrillary tangles [21], acts as both a contributing factor to and a consequence of neurodegeneration in AD [22,23]. In this study, we investigated whether oral administration of the ethanolic extract of EA leaves could alleviate neuroinflammation and further ameliorate major features of AD phenotypes in vivo.

The 5xFAD transgenic (Tg) mouse model expresses human Aβ precursor protein (APP) and presenilin-1 (PSEN1) transgenes with a total of five AD-linked mutations as follows: K670N/M671L (Swedish), I716V (Florida), and V717I (London) mutations in APP, and the M146L and L286V mutations in PSEN1 [17,24]. These mice develop age-dependent amyloid plaques, selective loss of noradrenergic and cholinergic neurons, memory impairments, and behavioral abnormalities [24,25]. This model offers several advantages, including the early onset of symptoms and, consequently, reduced housing costs [26]. Therefore, we employed the 5xFAD Tg mice to investigate the anti-AD potential of EA leaf extract.

## 2. Materials and Methods

### 2.1. EA Leaf Extract Preparation

EA leaves were supplied by the Forest Environment Research Institute of Gyeongsangbuk-do (Gyeongju, Republic of Korea). EA leaves (100 g) were extracted with 2 L of 80% (*v*/*v*) ethanolic solution combined with shaking (150 rpm) at 25 °C for 24 h. The extract was filtered using a filter paper (185 mm, Hyundai Micro, Seoul, Republic of Korea) and dehydrated by vacuum evaporation (EYELA N-1000, Tokyo, Japan), followed by freeze-drying. The dried sample was stored at −20 °C until use.

### 2.2. Experimental Animals

The 5xFAD Tg mice with a C57BL/6J genetic background were obtained from Jackson Laboratory (Bar Harbor, ME, USA) (strain: B6.Cg-Tg(APPSwFlLon,PSEN1*M146L*L286V)6799Vas/Mmjax; JAX MMRRC Stock #034848) [27]. These mice were bred with their congenic wild-type (WT) line, C57BL/6J female mice (Daehan Biolink, Eumseong, Republic of Korea). Following weaning, mice were co-housed with littermates under standard conditions (temperature, 20–25 °C; relative humidity, 45 ± 5%; light–dark cycle, 12 h:12 h) until the harvest dates. All mice had ad libitum access to a standard diet (Daehan BioLink, Eumseong, Republic of Korea). Male C57BL/6J WT mice were used as control animals.

### 2.3. Genotyping

Genomic DNA was extracted from the tail of mice for genotyping assay. The sample was incubated overnight in a solution containing TRI reagent (Sigma-Aldrich, St. Louis, MO, USA) and Proteinase K (RBC Bioscience, New Taipei City, Taiwan). The sample was vortexed after adding 200 μL of chloroform and subsequently centrifuged at 12,000× *g* for 5 min. After collecting the aqueous layer, 300 μL of 100% ethanol was added, followed by vortexing and subsequently centrifuging at 5000× *g* for 5 min. The DNA pellet was washed with 75% ethanol and air-dried. The DNA was dissolved in DNAase-free purified water and used as a template for genotyping. Genotyping was performed using the following primers: (1) 5′-ACCCCCATGTCAGAGTTCCT-3′ (common forward), (2) 5′-TATACAACCTTGGGGGATGG-3′ (WT reverse) for detecting the WT allele with an amplicon size of 216 bp, and (3) 5′-CGGGCCTCTTCGCTATTAC-3′ (mutant reverse) for detecting the mutant allele with an amplicon size of 129 bp.

### 2.4. Experimental Design for Animal Study

The animal study was conducted according to the guidelines of the Institutional Animal Care and Use Committee of Kyungpook National University (approval number: KNU 2022-0367). A total of 63 5xFAD Tg mice were randomly assigned to the seven groups (nine mice per group; Table 1). The groups were as follows: (1) Tg_Vehicle, Tg mice treated with vehicle only, (2) Tg_DPZ, Tg mice treated with donepezil (DPZ) at 5 mg/kg body weight (BW) per day, (3) Tg_EAE_L, Tg mice treated with EA leaf extract (EAE) at 50 mg/kg BW/day, (4) Tg_EAE_M, Tg mice treated with EAE at 100 mg/kg BW/day, (5) Tg_EAE_H, Tg mice treated with EAE at 150 mg/kg BW/day, (6) Tg_QCT_L, Tg mice treated with QCT at 1 mg/kg BW/day, and (7) Tg_QCT_H, Tg mice treated with QCT at 10 mg/kg BW/day. An additional experimental group of male C57BL/6J WT mice (referred to as ‘WT_Vehicle’) was used as a control (Table 1).

Mice were regularly orally administered DPZ, EAE, and QCT three times a week for 14 weeks. The vehicle used was saline containing 2% (*v*/*v*) Tween 80. BWs were monitored weekly throughout the study.

### 2.5. Behavioral Test

To assess the learning and memory abilities of 5xFAD mice, a behavioral test was performed using the passive avoidance task (PAT), Morris water maze task (MWMT), and Y-maze task (YMT) at the end of the treatment regimen as per the previously described procedures [28,29,30,31].

The PAT serves as a tool to assess fear-motivated learning and memory in mice. The apparatus (Gemini Avoidance System in San Diego, CA, USA) consisted of two chambers (dark and bright) separated by a guillotine door. On the initial test day, mice were placed in the bright chamber with the door open for 1 min to acclimate to the equipment. In the subsequent training trial, each mouse was placed in the bright chamber with a closed door. After 10 s, the door was opened, and when the mouse entered the dark chamber, the door closed. Following this, an electrical foot shock (0.5 mA, 3 s) was delivered through stainless steel rods in the dark chamber. The next day, during the test trial, each mouse was placed in the dark chamber with the door closed. After 10 s, the door opened, and the latency time spent in the dark chamber before escaping to the bright chamber was recorded. Prolonged latency time in the dark chamber was indicative of poor learning and memory abilities.

The MWM, conducted to assess spatial learning and memory, took place in a circular swimming pool (90 cm in diameter and 45 cm in height) with a featureless inner surface. The pool was filled with water (22 ± 2 °C) to a depth of 28 cm. On the first day, mice were allowed to swim freely for 1 min. Following this, a black platform (6 cm in diameter and 29 cm in height) was placed in one of the quadrants. The water was made opaque with non-toxic poster paint. Over the next three days, each mouse had three trials per session per day to locate the platform. If the mouse did not find the platform within 60 s, it was guided to the platform and allowed to stay for 10 s. On the fifth day, the platform was submerged 1 cm below the opaque water level in the same quadrant. The time each mouse spent swimming to locate the platform was recorded, analyzed, and graphed. Long arrival time at the platform was indicative of poor learning and memory abilities.

The YMT is a tool designed to explore working memory by assessing spontaneous alternation among the arms of a Y-shaped maze. A single test began by placing each mouse at the end of the A arm of the maze. The total number of arm entries and the sequence of entries were recorded over a 300 min period. Spontaneous alternations were defined as consecutive triplets of different arm choices. The percentage of alternations was calculated using the formula: % alternation = [(number of alternations)/(total arm entries − 2)] × 100.

### 2.6. Western Blot Analysis

The dissected brain tissues were prepared for extraction of cytoplasmic and nuclear proteins using the NE-PER^®^ nuclear and cytoplasmic extraction reagent (Thermo Fisher Scientific, Waltham, MA, USA) following the instructions by the supplier and as previously described [32,33]. Briefly, the tissues were manually homogenized in the appropriate volume of the CER I reagent containing the cOmplete™ Protease Inhibitor Cocktail (Roche, Basel, Switzerland) on ice. After adding the CER II reagent, the homogenates were incubated for 30 min with vigorous vortexing every 10 min and then centrifuged at 10,000× *g* for 5 min at 4 °C. The supernatant (cytoplasmic extract) was removed and stored in a pre-chilled tube. The pellet was suspended in an ice-cold NER reagent containing the protease inhibitor cocktail and incubated for 40 min with vigorous vortexing every 10 min. After centrifugation at 14,000× *g* for 10 min at 4 °C, the supernatant (nuclear extract) was removed and stored in a pre-chilled tube. The protein contents in the extracted fractions were quantified using Bradford assay [34]. Equal amounts of the proteins were denatured by heating at 95 °C for 10 min in a sample loading buffer for Western blotting (Sigma-Aldrich, St. Louis, MO, USA). The proteins were then electrophoretically separated on a 10% polyacrylamide gel and transferred onto the polyvinylidene difluoride membrane. Subsequently, antibody binding, visualization, and densitometry of protein bands on a gel were performed as previously described [35]. The primary antibodies used in this study were rabbit anti-COX-2 (Cell Signaling Technology, Danvers, MA, USA), rabbit anti-NF-κB (Cell Signaling Technology, Danvers, MA, USA), mouse anti-β-actin (Santa Cruz Biotechnology, Dallas, TX, USA), and goat anti-Lamin B (Santa Cruz Biotechnology, Dallas, TX, USA).

### 2.7. Oxidative Stress Level Determination

A byproduct of lipid peroxidation by oxidative stress was measured by the malondialdehyde (MDA) level in the tissue homogenate. Cerebral cortex tissues were collected from sacrificed animals, homogenized in a lysis buffer (0.1 M phosphate buffer, pH 7.4), and centrifuged at 10,000× *g* for 30 min at 4 °C. The supernatant was used for quantifying the content of thiobarbituric acid (TBA) reactive substances (TBARS) using the OXI-TEK TBARS Assay Kit (Cat. #ALX-850-287; Enzo Life Science, Inc., Farmingdale, NY, USA). Subsequently, 100 μL of the supernatant of the cerebrocortical tissue and the provided MDA standard were separately mixed with 100 μL of the sodium dodecyl sulfate-containing solution. Next, 2.5 mL of the TBA reagent was added and incubated at 95 °C for 60 min. The reactant was cooled down to room temperature on ice. After centrifugation, the supernatant was removed and subjected to measurement of the absorbance at a wavelength of 532 nm. Results were then normalized by the amount of protein determined using the Bradford assay.

The 8-hydroxydeoxyguanosine (8-OHdG) serves as a biomarker to identify DNA damage induced by oxidative stress. After completing the behavioral test, the plasma was extracted from the whole blood of each mouse and assessed for the level of 8-OHdG using DNA damage ELISA Kit (Cat. #ADI-EKS-350; Enzo Life Sciences, Inc.). Briefly, 50 μL of the plasma samples and the 8-OHdG standard were added to wells on the 8-OHdG immunoassay plate. Subsequently, 50 µL of anti-8-OHdG solution was added to those wells. The immunoassay plate was covered and incubated at room temperature for 2 h. The wells were washed with wash buffer, and 100 µL of anti-mouse IgG, HRP conjugate, was added to those wells. After incubation for 1 h, the wells were washed and allowed to react with the TMB substrate. After incubation for 15 min in the dark, the reaction was stopped by adding a Stop solution. The absorbance of the resultant reaction mixture was measured at 450 nm.

### 2.8. Hematoxylin and Eosin Staining

Brain tissues were dissected from the mice. Subsequently, the tissues were isolated, fixed in formalin, embedded in paraffin blocks, and sectioned at 5 µm thickness using a microtome (RM-2125 RT; Leica, Nussloch, Germany). These tissue sections were placed on microscope slides (Marienfeld, Lauda-Königshofen, Germany) and were subsequently stained with hematoxylin and eosin dyes (Sigma-Aldrich, St. Louis, MO, USA) as previously described [5,32]. The stained slices were mounted with a 1:1 solution of malinol medium (Muto Pure Chemical, Tokyo, Japan) and xylene.

### 2.9. Thioflavin S Staining

Tissue sections prepared on microscope slides were deparaffinized, rehydrated, and immersed in a solution of 0.002% (*w*/*v*) thioflavin S (Sigma-Aldrich, St. Louis, MO, USA) and 50% ethanol. Following sequential rinsing with 50% ethanol and PBS, nuclei were counterstained with 4′,6-diamidino-2-phenylindole dihydrochloride (DAPI). The sections were mounted using a commercially available mounting medium (Dako Denmark A/S, Glostrup, Denmark). The thioflavin S–labeled Aβ plaques and DAPI-stained nuclei were observed under a fluorescence microscope (Eclipse 80i, Nikon, Tokyo, Japan) at 100× magnification.

### 2.10. Statistical Analysis

The obtained data were expressed as means ± standard deviations (SDs) and analyzed by one-way analysis of variance, followed by Duncan’s multiple range test, using SPSS software version 25.0 (IBM SPSS Statistics, Chicago, IL, USA). Statistical differences were considered significant at *p*-values < 0.05 and were indicated using distinct alphabetical letters.

## 3. Results

### 3.1. EAE Treatment Improved Learning and Memory Abilities in 5xFAD Mice

**Figure 1:**
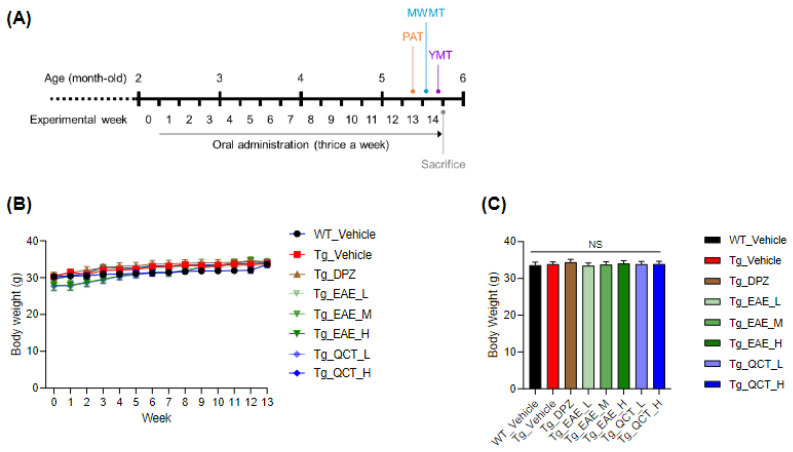
Animal experimental scheme. (**A**) Euonymus alatus leaf extract (EAE) was orally administered to 2-month-old 5xFAD mice (nine mice per group) at the designated doses thrice a week for 14 weeks. (**B**) The body weight (BW) of each experimental mouse was regularly monitored during the entire experimental period. (**C**) The averaged BWs at the termination of the experiment were graphed, and no significant difference was observed among the groups.

Starting from 2 months old, 5xFAD mice were orally administered thrice a week for 14 weeks (Figure 1A). The experimental groups comprised a group of WT mice treated with vehicle only (‘WT_Vehicle’) and seven groups of Tg mice as follows: Tg_Vehicle, Tg_DPZ (5 mg/kg BW/day), Tg_EAE_L (50 mg/kg BW/day), Tg_EAE_M (100 mg/kg BW), Tg_EAE_H (150 mg/kg BW/day), Tg_QCT_L (1 mg/kg BW/day), and Tg_QCT_H (10 mg/kg BW/day) (Table 1). The BWs of all experimental mice were regularly measured. There were no significant differences in average BW among the groups (Figure 1B,C), indicating that oral administration of EAE and QCT was non-toxic at the tested doses.

**Figure 2:**
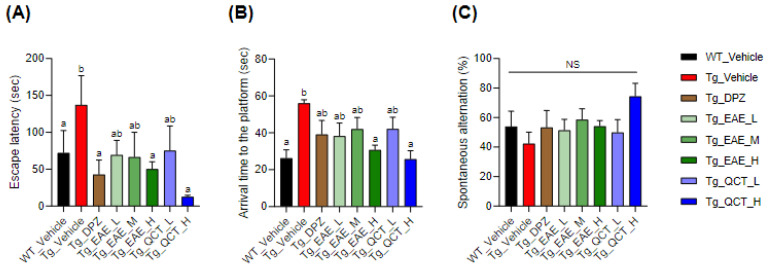
EAE treatment improved memory-associated behaviors in 5xFAD mice. 5xFAD mice were fed with either EAE at 50, 100, and 150 mg/kg BW/day or quercetin (QCT) at 1 and 10 mg/kg BW/day thrice weekly for 14 weeks. (**A**–**C**) The behavioral test was performed using the passive avoidance task (**A**), Morris water maze task (**B**), and Y-maze task (**C**). Values are expressed as means ± standard deviations (SDs) (*n* = 9). Different letters indicate significant differences among treatments.

At the end of the treatment regimen, a comprehensive behavioral assessment of learning and memory was conducted as scheduled (Figure 2). This included the PAT (to evaluate the ability of associative memory by contextual fear conditioning), MWMT (to evaluate spatial learning and memory), and YMT (to evaluate working memory driven by an innate curiosity of mice to explore previously unvisited areas). The PAT and MWMT results revealed that treatment with EAE at 150 mg/kg BW/day and QCT at 10 mg/kg BW/day significantly improved associative and spatial learning and memory in 5xFAD mice (Figure 2A,B). However, no significant difference in working memory improvement was observed among any of the experimental groups (Figure 2C).

### 3.2. EAE Treatment Decreased Inflammatory Mediator Levels in 5xFAD Mice

**Figure 3:**
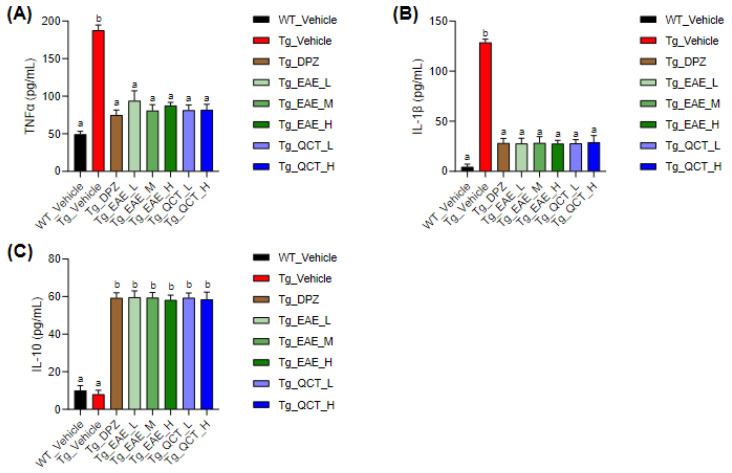
EAE treatment reduced inflammation-related cytokines in the plasma of 5xFAD mice. At the termination of the experiment, blood was collected from the mice, and plasma was obtained following centrifugation in the presence of heparin. (**A**–**C**) Plasma levels of inflammation-related cytokines were determined using an ELISA assay. The levels of inflammatory cytokines, TNFα (**A**) and IL-1β (**B**), and anti-inflammatory cytokine, IL-10 (**C**), were determined. Values are expressed as means ± SDs (*n* = 9). Different letters indicate significant differences among treatments.

The levels of inflammatory mediators were examined to determine a possible mechanism of learning and memory improvement following oral administration of EAE. Compared with the Tg_Vehicle group, the plasma levels of pro-inflammatory cytokines, TNFα and IL-1β, were significantly decreased in all groups treated with either EAE or QCT regardless of doses (Figure 3A,B). Additionally, EAE and QCT treatment significantly increased the level of IL-10, an anti-inflammatory cytokine mediating neuroprotection, in 5xFAD mice compared with untreated Tg mice (Figure 3C).

**Figure 4:**
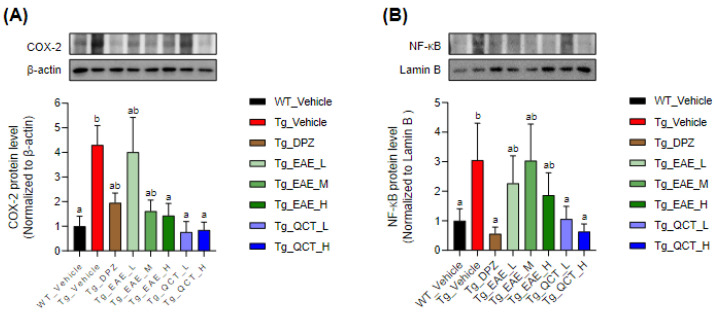
EAE treatment decreased the expressions of inflammatory markers in the hippocampus of 5xFAD mice. (**A**,**B**) Expression levels of cytoplasmic COX-2 (**A**) and nuclear NF-κB proteins (**B**) in the hippocampal tissue homogenates were assessed using Western blot analysis. Values are expressed as means ± SDs (*n* = 6). Different letters indicate significant differences among treatments.

Moreover, 5xFAD Tg mice showed significantly higher levels of cytoplasmic COX-2 and nuclear NF-κB proteins in their hippocampal homogenates than WT mice (Figure 4). Treatment with QCT at ≥1 mg/kg BW/day markedly decreased cytoplasmic COX-2 and nuclear NF-κB levels in the hippocampi of 5xFAD mice. Notably, EAE treatment at 150 mg/kg BW/day only decreased COX-2 expression without significantly affecting nuclear NF-κB levels. These results suggested that EAE or QCT treatment over a relatively long period alleviated inflammatory response in the brain, particularly the hippocampal region.

### 3.3. EAE Treatment Decreased Oxidative Stress Marker Levels in 5xFAD Mice

The 14-week oral administration of either EAE or QCT in 5xFAD mice at 2–6 months old markedly decreased 8-OHdG levels in the plasma and MDA levels in the cortex of the brain (Supplementary Figure S1). These observations suggested that the mitigation of learning and memory deficits following EAE or QCT treatment of 5xFAD mice would be attributed to their combined antioxidative and anti-inflammatory effects in vivo.

### 3.4. EAE Treatment Decreased Aβ Accumulation in the Hippocampus of 5xFAD Mice

**Figure 5:**
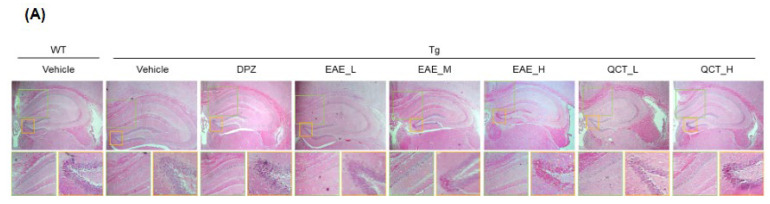

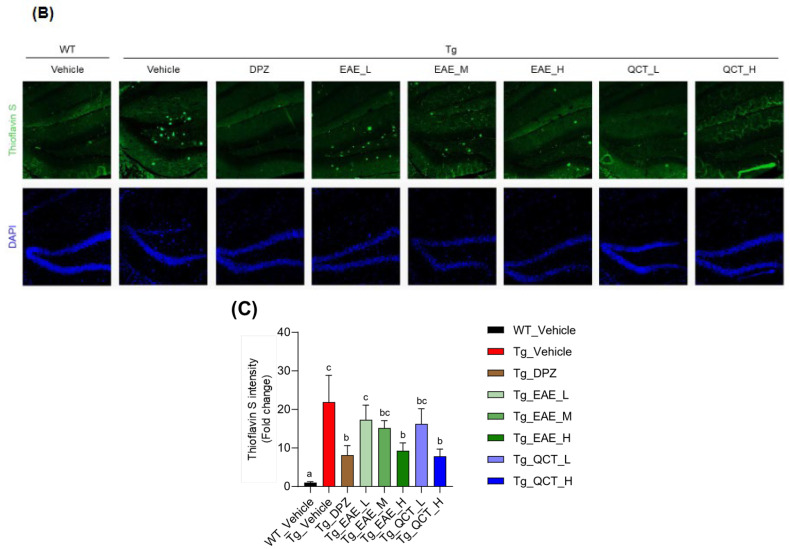
EAE treatment ameliorated histological damage and Aβ accumulation in the hippocampal area of 5xFAD mice. (**A**) Representative images of brain slices stained by hematoxylin and eosin. Upper panel, hippocampal area of the mouse brain. Lower left panel (boxed in green), CA1 region. Lower right panel (boxed in yellow), DG region. (**B**,**C**) Hippocampal region stained by thioflavin S. Representative images at 100× magnification (**B**) and quantification data for thioflavin S-staining intensity (**C**).

Histological analysis was performed to determine whether EAE and QCT can prevent hippocampal damage, which gradually occurs with aging in 5xFAD mice (Figure 5). EAE treatment considerably improved the histological abnormalities in the hippocampal CA1 and DG regions (Figure 5A) and reduced the Aβ plaques that accumulated in the hippocampal area profoundly observed in the 5xFAD mouse brain (Figure 5B,C). These results indicated that EAE treatment suppressed hippocampal damage and Aβ aggregation and thereby may improve cognitive deficits in 5xFAD mice.

## 4. Discussion

In this study, we examined the anti-AD potential of a long-term EAE intake in 5xFAD Tg mice, a model of AD. The 5xFAD mouse, which highly expresses mutant human APP and PSEN1, is frequently used for investigating AD pathology in vivo, including amyloid plaque formation and cognitive impairment. Two-month-old mice were orally administered with a vehicle, DPZ (at 5 mg/kg BW/day as a positive control), EAE (at 50, 100, or 150 mg/kg BW/day), or QCT (1 or 10 mg/kg BW/day) three times a week for 14 weeks, and their behavioral properties and tissue levels of inflammatory mediators were subsequently examined. We observed that EAE treatment at 150 mg/kg BW/day or QCT at 10 mg/kg BW/day alleviated learning and memory decline through, at least in part, inflammatory response and oxidative damage attenuation in 5xFAD mice.

Our previous study demonstrated that EAE exhibited a radical scavenging capacity, antioxidant enzyme-inducing activity in vitro, and a neuroprotective effect in scopolamine-induced acute amnesia in a normal mouse model [5]. In line with these earlier findings, we noted the anti-inflammatory and anti-AD potential of the oral administration of EAE to 5xFAD AD mice for a relatively extended period. This potential was evidenced by a significant decrease in inflammatory responses and a concurrent amelioration in learning and memory impairments.

Neuroinflammation is a key event in the central nervous system in AD pathophysiology [22]. Several neuroinflammatory factors are involved in both AD onset and progression. This process depends on the innate immune system, including microglia and astrocytes [36]. Aβ oligomers can induce microglial activation through cell-surface receptors, including toll-like receptors (TLRs). Astrocytes play roles in the clearance of Aβ by enzyme secretion as well as metabolic regulation, neuronal scaffold, and synaptogenesis. Astrocytes surrounding Aβ plaques turn into an activated form through a pathway involving the NF-κB. The activated astrocytes exhibit intracellular calcium deregulation, defect in Aβ clearance, and release of complement C3, which consequently induces neuronal damage [16,21,22]. Our results demonstrated that EAE treatment decreased the nuclear level of NF-κB, a key immune and inflammatory response regulator, and the cytoplasmic expression level of one of its downstream proteins, COX-2, in the hippocampal tissue of the 5xFAD mouse brain. It suggests that a long-term EAE intake can suppress inflammatory responses in the AD brain; however, the pathophysiological mechanism of action remains to be elucidated.

Several studies have shown that rutin (quercetin-3-*O*-rutinose) is abundantly present in EA leaves and stalks, and free QCT can induce defensive antioxidant enzymes in an Nrf2-dependent manner [4,37]. Recently, it has been reported that QCT can reduce inflammation and protect against neuroinflammatory toxicity in vitro and in vivo models [38,39,40]. Regarding bioavailability, QCT can be easily maintained at a high concentration in plasma when supplied in the diet and also directly absorbed in the small and/or large intestine without further hydrolysis [41,42]. In this study, we measured the quantities of QCT in EA leaves and EAE, finding approximately 0.724 mg QCT per gram of dry leaf matter and 62.245 mg QCT per gram of EAE. Thus, the administration of EAE at 150 mg/kg BW/day was calculated to be equivalent to the administration of QCT at 9.337 mg/kg BW/day. Therefore, the observed anti-inflammatory effect of EAE in this study was likely, at least partially, due to the presence of QCT in EAE. However, further research is needed to determine the bioavailability of QCT or other bioactive components and their specific contributions to the anti-AD potential of EAE.

Overall, the findings from this study suggest that a long-term EAE administration containing a rational quantity of QCT can alleviate the major features of AD phenotypes and delay their progression.

## 5. Conclusions

Our study explored the potential of long-term EAE intake in mitigating AD features using the 5xFAD Tg mouse model. Administering EAE at 150 mg/kg BW/day or QCT at 10 mg/kg BW/day demonstrated promising results, alleviating learning and memory decline, potentially through the attenuation of inflammatory responses and oxidative damage. The observed reduction in NF-κB and COX-2 levels in the hippocampal tissue suggests that EAE may effectively suppress neuroinflammatory pathways in the AD brain. The presence of QCT in EAE, known for its anti-inflammatory properties, may contribute to these effects. However, a comprehensive understanding of bioavailability and the specific role of QCT in the anti-AD potential of EAE requires further investigation. Therefore, our findings position EAE as a candidate for future therapeutic interventions targeting AD-associated pathologies and cognitive impairments.

## Figures and Tables

**Table 1:** Experimental groups for the animal study using 5xFAD mice.

Group	Experimental Groups (*n* = 9 Mice/Group)							
	1	2	3	4	5	6	7	8
Mouse type	WT (C57BL/6J)	5xFAD Tg						
EA treatment (*p.o.*^(1)^)	Vehicle	Vehicle	Donepezil	EAE ^(2)^			QCT ^(3)^	
	-	-	5 mg/kg BW/day	50 mg/kg BW/day	100 mg/kg BW/day	150 mg/kg BW/day	1 mg/kg BW/day	10 mg/kg BW/day

^(1)^ *p.o.*, *per os*, oral administration. ^(2)^ EAE, EA extract. ^(3)^ QCT, quercetin.

## Data Availability

The authors declare that all data supporting the findings of this study are available in the article and can be provided by the corresponding author upon reasonable request.

## Supplementary Materials

The following supporting information can be downloaded at: https://www.mdpi.com/article/10.3390/foods13050682/s1. **Figure S1.** EAE treatment decreased oxidative stress marker levels in 5xFAD mice. (A–B) Levels of oxidative stress markers 8-hydroxydeoxyguanosine (8-OHdG) in the plasma (A) and malondialdehyde (MDA) in the cerebral cortex (B). Values are expressed as means ± SDs (*n* = 6). Different letters indicate significant differences among treatments. **Figure S2.** Western blot images of the graphs in Figure 4. Hippocampal tissues were dissected from all of experimental mice. Tissue homogenates were used to determine the expression levels of COX-2 (A), β-actin (B), NF-κB (C), and lamin B (D).

## References

[1] Fan L., Zhang C., Ai L., Wang L., Li L., Fan W., Li R., He L., Wu C., Huang Y. (2020). Traditional uses, botany, phytochemistry, pharmacology, separation and analysis technologies of *Euonymus alatus* (Thunb.) Siebold: A comprehensive review. J. Ethnopharmacol..

[2] Kwon G.-J., Choi D.-S., Wang M.-H. (2007). Biological activities of hot water extracts from *Euonymus alatus* leaf. Korean J. Food Sci. Technol..

[3] Park S.H., Ko S.K., Chung S.H. (2005). *Euonymus alatus* prevents the hyperglycemia and hyperlipidemia induced by high-fat diet in ICR mice. J. Ethnopharmacol..

[4] Fang X.-K., Gao J., Zhu D.-N. (2008). Kaempferol and quercetin isolated from *Euonymus alatus* improve glucose uptake of 3T3-L1 cells without adipogenesis activity. Life Sci..

[5] Woo Y., Lim J.S., Oh J., Lee J.S., Kim J.S. (2020). Neuroprotective Effects of *Euonymus alatus* Extract on Scopolamine-Induced Memory Deficits in Mice. Antioxidants.

[6] Kim G.H., Kim J.E., Rhie S.J., Yoon S. (2015). The Role of Oxidative Stress in Neurodegenerative Diseases. Exp. Neurobiol..

[7] Niedzielska E., Smaga I., Gawlik M., Moniczewski A., Stankowicz P., Pera J., Filip M. (2016). Oxidative Stress in Neurodegenerative Diseases. Mol. Neurobiol..

[8] Olufunmilayo E.O., Gerke-Duncan M.B., Holsinger R.M.D. (2023). Oxidative Stress and Antioxidants in Neurodegenerative Disorders. Antioxidants.

[9] Sinyor B., Mineo J., Ochner C. (2020). Alzheimer’s Disease, Inflammation, and the Role of Antioxidants. J. Alzheimer’s Dis. Rep..

[10] Tonnies E., Trushina E. (2017). Oxidative Stress, Synaptic Dysfunction, and Alzheimer’s Disease. J. Alzheimer’s Dis. JAD.

[11] Jayaram S., Krishnamurthy P.T. (2021). Role of microgliosis, oxidative stress and associated neuroinflammation in the pathogenesis of Parkinson’s disease: The therapeutic role of Nrf2 activators. Neurochem. Int..

[12] Mosley R.L., Benner E.J., Kadiu I., Thomas M., Boska M.D., Hasan K., Laurie C., Gendelman H.E. (2006). Neuroinflammation, Oxidative Stress and the Pathogenesis of Parkinson’s Disease. Clin. Neurosci. Res..

[13] DeTure M.A., Dickson D.W. (2019). The neuropathological diagnosis of Alzheimer’s disease. Mol. Neurodegener..

[14] Long J.M., Holtzman D.M. (2019). Alzheimer Disease: An Update on Pathobiology and Treatment Strategies. Cell.

[15] Jorfi M., Maaser-Hecker A., Tanzi R.E. (2023). The neuroimmune axis of Alzheimer’s disease. Genome Med..

[16] Zhang Y., Chen H., Li R., Sterling K., Song W. (2023). Amyloid beta-based therapy for Alzheimer’s disease: Challenges, successes and future. Signal Transduct. Target. Ther..

[17] Yun H.S., Oh J., Lim J.S., Kim H.J., Kim J.S. (2021). Anti-Inflammatory Effect of Wasp Venom in BV-2 Microglial Cells in Comparison with Bee Venom. Insects.

[18] Cacabelos R., Carrera I., Martínez-Iglesias O., Cacabelos N., Naidoo V. (2021). What is the gold standard model for Alzheimer’s disease drug discovery and development?. Expert Opin. Drug Discov..

[19] Kimura R., Ohno M. (2009). Impairments in remote memory stabilization precede hippocampal synaptic and cognitive failures in 5XFAD Alzheimer mouse model. Neurobiol. Dis..

[20] Kinney J.W., Bemiller S.M., Murtishaw A.S., Leisgang A.M., Salazar A.M., Lamb B.T. (2018). Inflammation as a central mechanism in Alzheimer’s disease. Alzheimers Dement.

[21] Zhang W., Xiao D., Mao Q., Xia H. (2023). Role of neuroinflammation in neurodegeneration development. Signal Transduct. Target. Ther..

[22] Guzman-Martinez L., Maccioni R.B., Andrade V., Navarrete L.P., Pastor M.G., Ramos-Escobar N. (2019). Neuroinflammation as a Common Feature of Neurodegenerative Disorders. Front. Pharmacol..

[23] Ransohoff R.M. (2016). How neuroinflammation contributes to neurodegeneration. Science.

[24] Jawhar S., Trawicka A., Jenneckens C., Bayer T.A., Wirths O. (2012). Motor deficits, neuron loss, and reduced anxiety coinciding with axonal degeneration and intraneuronal Aβ aggregation in the 5XFAD mouse model of Alzheimer’s disease. Neurobiol. Aging.

[25] Kosel F., Munoz P.T., Yang J.R., Wong A.A., Franklin T.B. (2019). Age-related changes in social behaviours in the 5xFAD mouse model of Alzheimer’s disease. Behav. Brain Res..

[26] Bilkei-Gorzo A. (2014). Genetic mouse models of brain ageing and Alzheimer’s disease. Pharmacol. Ther..

[27] Neuner S.M., Heuer S.E., Huentelman M.J., O’Connell K.M., Kaczorowski C.C. (2019). Harnessing genetic complexity to enhance translatability of Alzheimer’s disease mouse models: A path toward precision medicine. Neuron.

[28] Lee S., Lim J.S., Yun H.S., Kim Y., Jeong S., Hwang S.D., Kim J.W., Oh J., Kim J.S. (2021). Dietary supplementation with Ceriporia lacerata improves learning and memory in a scopolamine-induced amnesia mouse model. Food Sci. Biotechnol..

[29] Kim M.H., Seo J.Y., Kim J.S. (2015). *Artemisia annua* L. extract ameliorates galactose-induced cognitive impairment in mice. Food Sci. Biotechnol..

[30] Seo J.Y., Ju S.H., Oh J., Lee S.K., Kim J.S. (2016). Neuroprotective and Cognition-Enhancing Effects of Compound K Isolated from Red Ginseng. J. Agric. Food Chem..

[31] Kim S., Oh J., Jang C.H., Kim J.S. (2019). Improvement of cognitive function by Gochujang supplemented with tomato paste in a mouse model. Food Sci. Biotechnol..

[32] Jeong Y.A., Yun H.S., Kim Y., Jang C.H., Lim J.S., Kim H.J., Choi M.B., Jung J.W., Oh J., Kim J.S. (2023). Long-Term Administration of Vespa velutina nigrithorax Venom Ameliorates Alzheimer’s Phenotypes in 5xFAD Transgenic Mice. Toxins.

[33] Seo H., Oh J., Hahn D., Kwon C.S., Lee J.S., Kim J.S. (2017). Protective Effect of Glyceollins in a Mouse Model of Dextran Sulfate Sodium-Induced Colitis. J. Med. Food.

[34] Bradford M.M. (1976). A rapid and sensitive method for the quantitation of microgram quantities of protein utilizing the principle of protein-dye binding. Anal. Biochem..

[35] Weiss W., Vogelmeier C., Görg A. (1993). Electrophoretic characterization of wheat grain allergens from different cultivars involved in bakers’ asthma. Electrophoresis.

[36] Maccioni R.B., Rojo L.E., Fernandez J.A., Kuljis R.O. (2009). The role of neuroimmunomodulation in Alzheimer’s disease. Ann. N. Y. Acad. Sci..

[37] Zhang F., Yang Y., Su P., Guo Z. (2009). Microwave-assisted extraction of rutin and quercetin from the stalks of *Euonymus alatus* (Thunb.) Sieb. Phytochem. Anal. PCA.

[38] Khan A., Ali T., Rehman S.U., Khan M.S., Alam S.I., Ikram M., Muhammad T., Saeed K., Badshah H., Kim M.O. (2018). Neuroprotective Effect of Quercetin Against the Detrimental Effects of LPS in the Adult Mouse Brain. Front. Pharmacol..

[39] Han X., Xu T., Fang Q., Zhang H., Yue L., Hu G., Sun L. (2021). Quercetin hinders microglial activation to alleviate neurotoxicity via the interplay between NLRP3 inflammasome and mitophagy. Redox Biol..

[40] Chiang M.C., Tsai T.Y., Wang C.J. (2023). The Potential Benefits of Quercetin for Brain Health: A Review of Anti-Inflammatory and Neuroprotective Mechanisms. Int. J. Mol. Sci..

[41] Manach C., Morand C., Demigne C., Texier O., Regerat F., Remesy C. (1997). Bioavailability of rutin and quercetin in rats. FEBS Lett..

[42] Santos M.R., Rodriguez-Gomez M.J., Justino G.C., Charro N., Florencio M.H., Mira L. (2008). Influence of the metabolic profile on the in vivo antioxidant activity of quercetin under a low dosage oral regimen in rats. Br. J. Pharmacol..

