# Supplementary material for: Oral Administration of Euonymus alatus Leaf Extract Ameliorates Alzheimer’s Disease Phenotypes in 5xFAD Transgenic Mice
Source: Foods. 2024 Feb 23;13(5):682. doi: 10.3390/foods13050682 (PMC10930996; doi:10.3390/foods13050682)

Supplementary information

Supplementary Figure S1

(A)

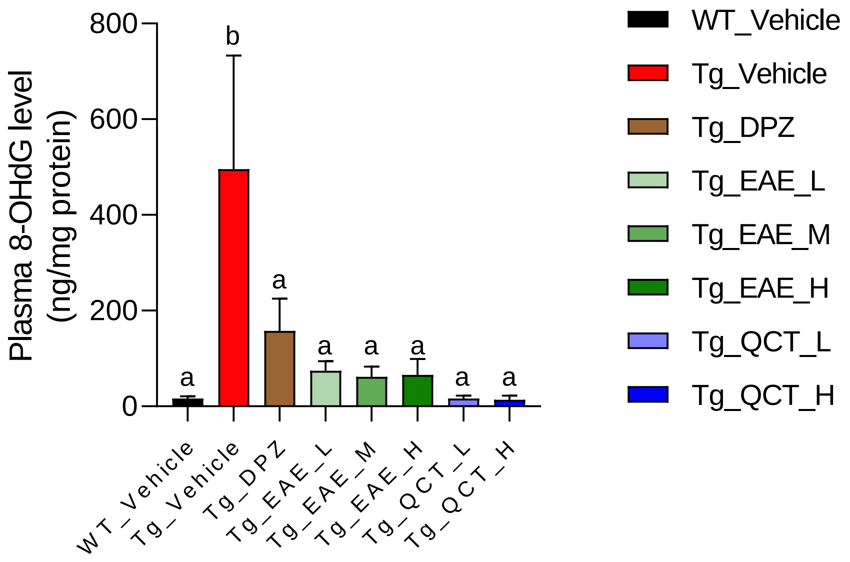

(B)

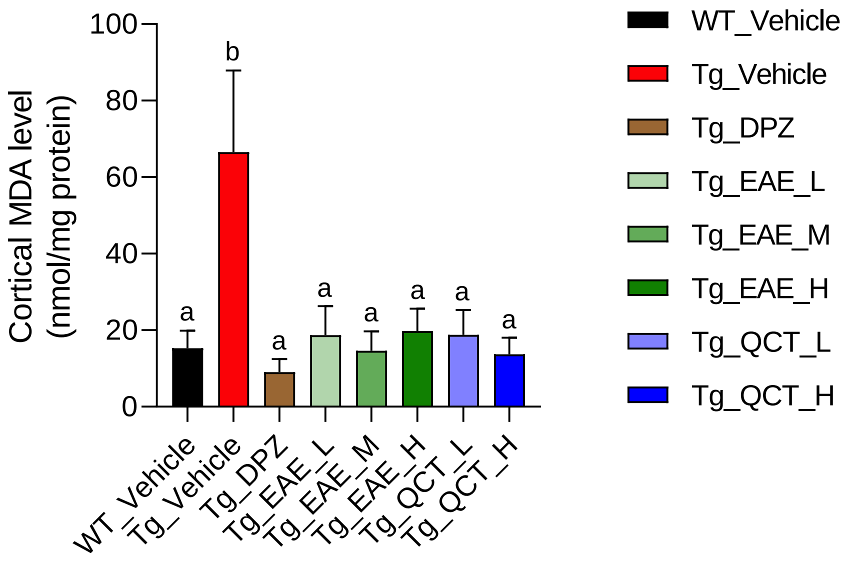

Supplementary Figure S2

(A) COX-2 (74 kD)

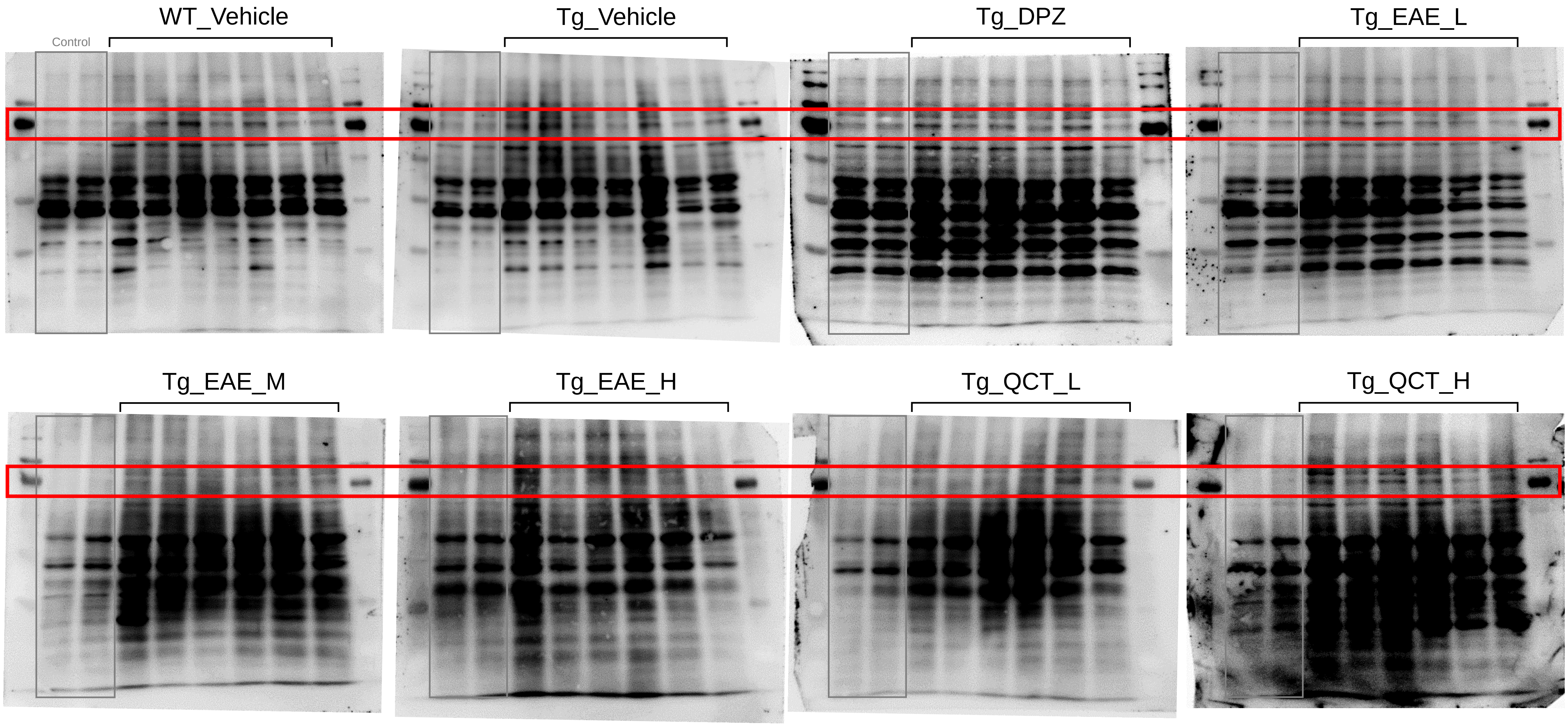

(B) β-actin (43 kD)

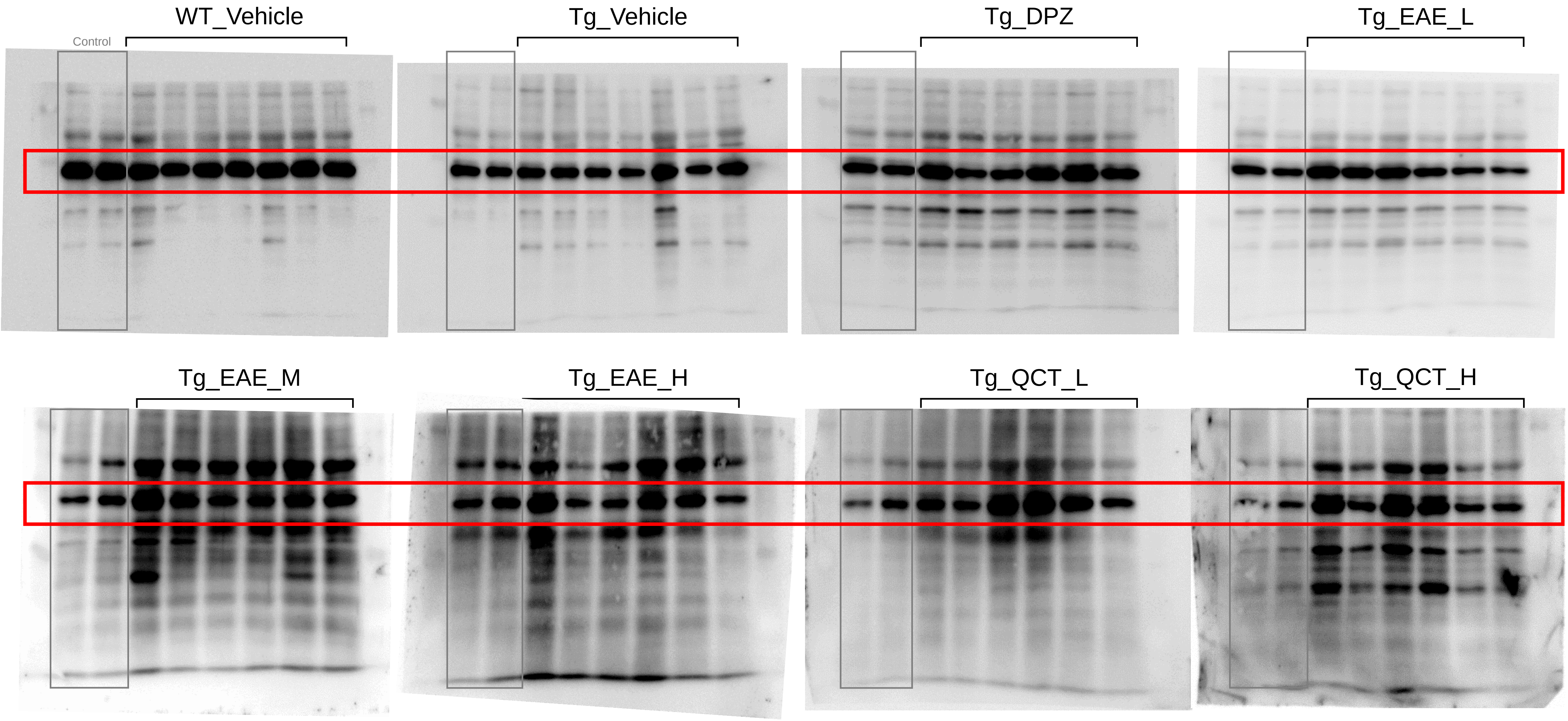

(C) NF-κB (65 kD)

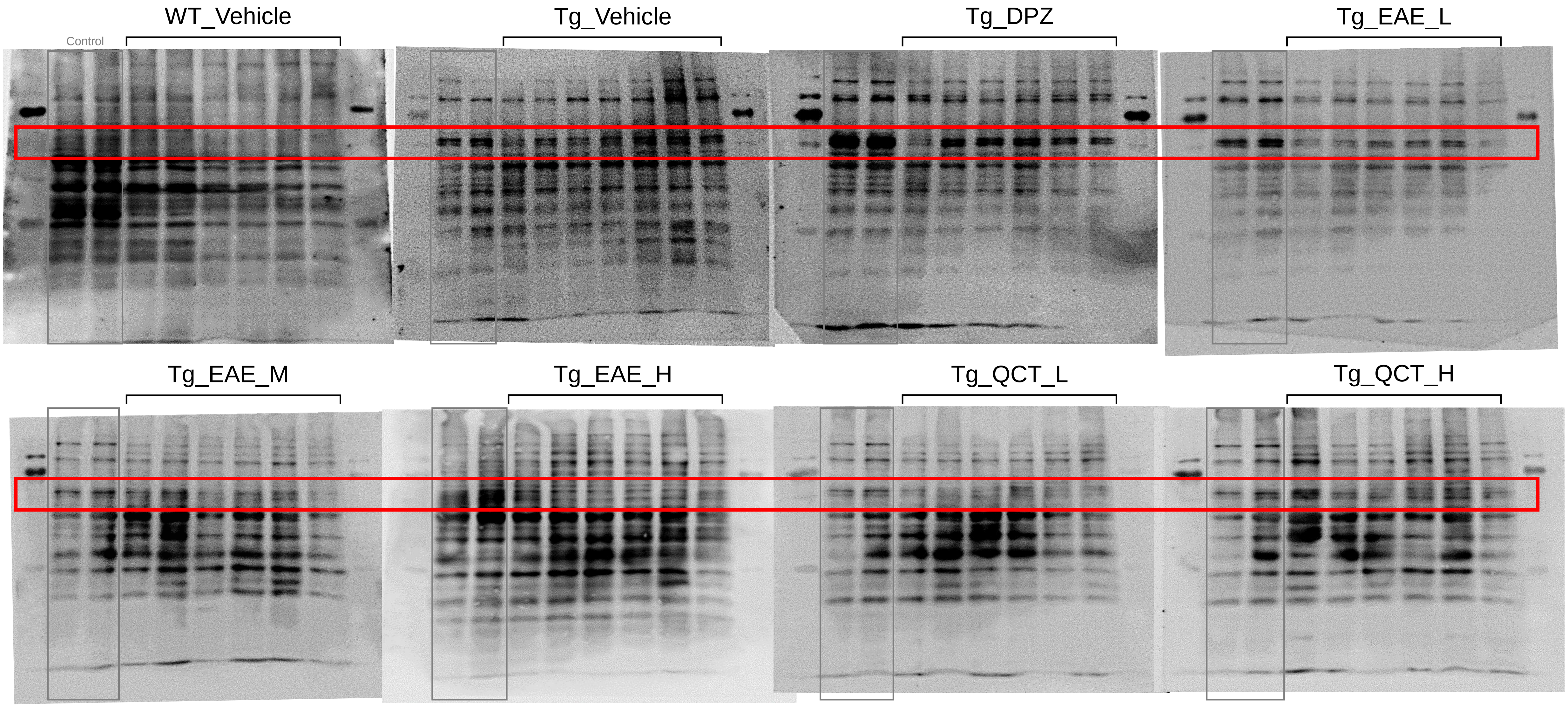

WT_Vehicle
Control
Tg_Vehicle
Tg_DPZ
Tg_EAE_L
Tg_EAE_M
Tg_EAE_H
Tg_QCT_L
Tg_QCT_H

(D) Lamin B (67 kD)

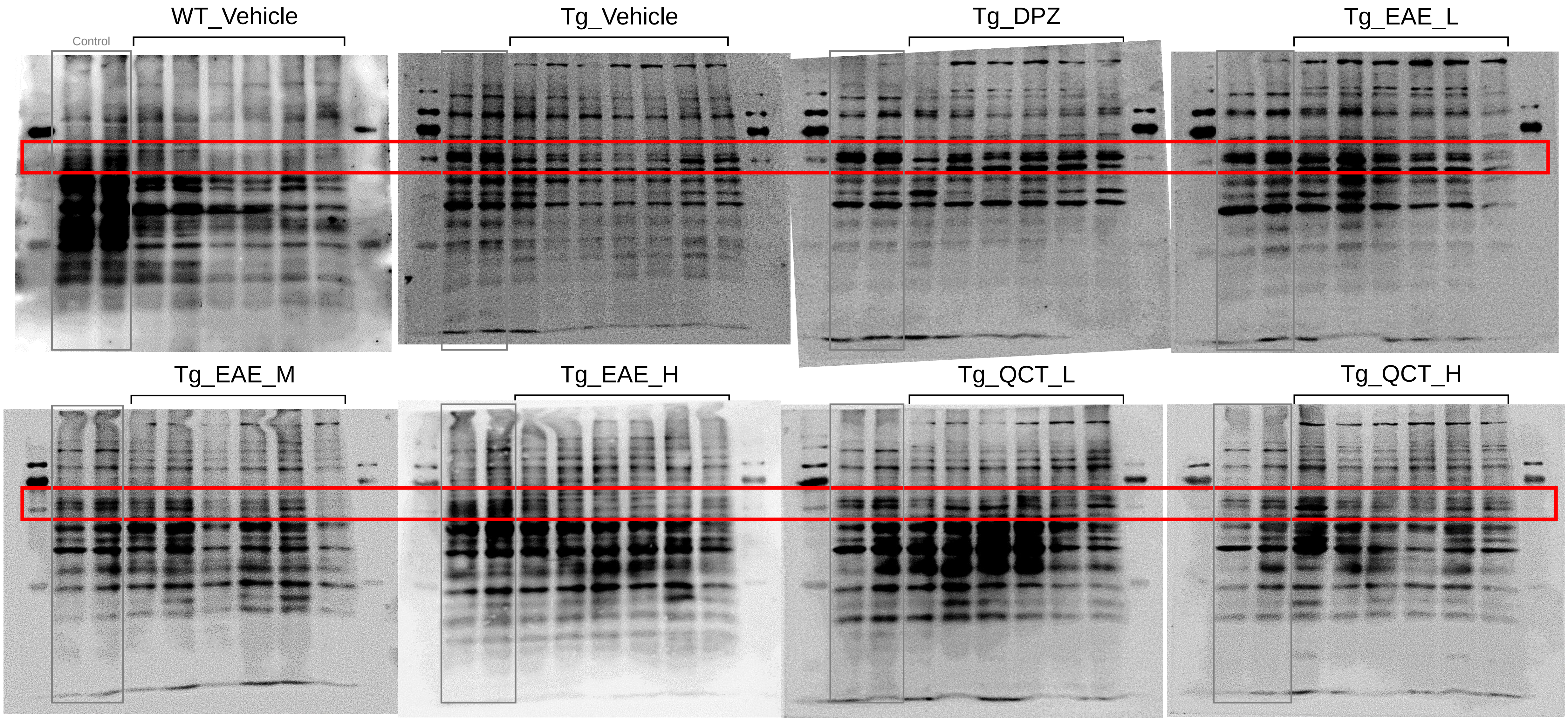

Supplement: Supplementary file 1 [file foods-13-00682-s001.zip › foods-2819099-supplementary materials.pdf]
